# Does lateral whole-spine radiograph taken by fists-on-clavicle position represent reliable global sagittal spinal alignment in adolescent patients?

**DOI:** 10.1186/1748-7161-10-S1-O41

**Published:** 2015-01-19

**Authors:** Futoshi Asano, Yuichiro Abe, Shigenobu Sato, Tetsuya Kobayashi, Shizuo Jinbo

**Affiliations:** 1 Departments of Orthopaedics, Eniwa Hospital, Japan; 2 Departments of Orthopaedics, Asahikawa Medical College, Japan

## Introduction

Evaluation of global sagittal spinal alignment has been recognized to be important for treating spinal deformity in recent years. Global sagittal alignment was evaluated on the lateral whole-spine radiograph and "fists-on-clavicle position" was widely used as arm position during radiographic acquisition. Several studies reported the validity of the fits-on-clavicle position in adult patients, but there were few studies about adolescent patients due to the radiation exposure problem. In the present study, we evaluated postural difference between natural drop arm and fits-on-clavicle position using radiation free 3D scanner system.

## Material and method

A total of 24 adolescent patients (8boys, 16girls) who were taken 3D-photograph of body surface of the back in school physical examination of scoliosis were enrolled in the study. Averaged age at examination was 11.9 years old. Shape of the body surface was collected using 3D projection scanning system (SLS-1, David Vision). Scans were taken in two different postures; natural drop arm position (NP) and fists-on-clavicle position (CP). Shift of C7 spinous process in sagittal plane, thoracic kyphosis and lumbar lordosis were evaluated using originally developed software (ZedView VEGA, LEXI).

## Results

Posterior shift of C7 was observed in CP compared with NP in all 24 patients and mean change (NP to CP) was 24.7±15mm. 38% of patients showed significant posterior plumb line shift over 30mm from NP to CP. Change in thoracic kyphosis from NP to CP was -3.1±5.4 degrees and thoracic kyphosis was significantly smaller in CP compared with NP (39.9, 43.0 degrees, respectively). Change in lumbar lordosis from NP to CP was +2.7±3.4 degrees and lumbar lordosis was significantly larger in CP compared with NP (40.4, 37.8 degrees, respectively).

## Conclusions

All patient showed negative shift of sagittal vertical axis (SVA) in fits-on-clavicle position. This postural change of increase in lumbar lordosis and decrease in thoracic kyphosis with posterior trunk shift is called "sway back position", and this postural change is known to induce apparent decrease in cervical lordosis. To obtain reliable lateral whole-spine radiograph, sway back phenomenon must be kept to the minimum, and we should keep in mind that lateral radiograph could represent negative SVA position in adolescent patients.

